# Impact of Obesity on Chemotherapy Pharmacokinetics, Toxicity, and Survival Outcomes in Pediatric Acute Lymphoblastic Leukemia

**DOI:** 10.7759/cureus.116568

**Published:** 2026-09-20

**Authors:** Arun Nair, Gillian Graifman, U Tahir, Riddhi Modi, Ranya Parekh, Rajesh Varma CP, Akash Khanal

**Affiliations:** 1 Pediatric Hematology Oncology, Cohen Children's Medical Center, New Hyde Park, USA; 2 Pediatrics, Cohen Children's Medical Center, New Hyde Park, USA; 3 Epidemiology and Biostatistics, The City University of New York (CUNY) Graduate School of Public Health and Health Policy, New York City, USA; 4 General Practice, Jagadguru Sri Shivarathreeshwara (JSS) University, Mysore, IND; 5 Medicine, Donald and Barbara Zucker School of Medicine at Hofstra/Northwell, Hempstead, USA; 6 Pediatrics, Saint Peter's University Hospital, New Brunswick, USA

**Keywords:** acute lymphoblastic leukemia (all), clinical pharmacokinetics, methotrexate, obesity, pediatric oncology, pegaspargase

## Abstract

Obesity is increasingly prevalent among children and adolescents and may influence chemotherapy pharmacokinetics, treatment-related toxicity, and clinical outcomes in pediatric acute lymphoblastic leukemia (ALL). This narrative review evaluates current evidence regarding the effects of obesity on drug disposition, chemotherapy-associated toxicity, dosing considerations, and survival outcomes in pediatric ALL. Obesity alters body composition and may affect hepatic and renal physiology; however, its pharmacokinetic effects vary among therapeutic agents. Studies demonstrate associations between greater body size or adiposity and delayed high-dose methotrexate elimination, modestly reduced pegaspargase clearance, and lower thioguanine nucleotide concentrations during mercaptopurine maintenance, although these findings have not consistently translated into differences in systemic exposure or treatment efficacy. Obesity has also been associated with hepatotoxicity, pancreatitis, conjugated hyperbilirubinemia, and vincristine-induced peripheral neuropathy. Importantly, persistent overweight or obesity and extreme obesity have been associated with inferior survival outcomes or increased relapse risk in some cohorts, although the mechanisms underlying these associations remain incompletely understood. Current evidence does not support generalized chemotherapy dose reduction based solely on obesity. Instead, management should remain drug- and protocol-specific and incorporate organ function, pharmacogenetics, therapeutic drug monitoring when available, and observed toxicity. Prospective studies integrating longitudinal body composition with pharmacokinetic, pharmacodynamic, toxicity, and survival measures are needed to define whether specific patient subgroups may benefit from individualized dosing strategies.

## Introduction and background

Acute lymphoblastic leukemia (ALL) is a hematologic malignancy characterized by clonal proliferation of immature lymphoid precursor cells, primarily in the bone marrow, with variable involvement of peripheral blood and extramedullary sites [1]. It is the most common childhood malignancy and the predominant form of pediatric leukemia [2]. In the United States, approximately 3000 children and adolescents are diagnosed annually, with incidence between two and four years of age [2]. Advances in risk-adapted, multiagent therapy have substantially improved prognosis, with overall survival approaching or exceeding 90% in contemporary pediatric populations [3].

Pediatric ALL is classified according to lymphoid lineage into B-cell acute lymphoblastic leukemia (B-ALL) or T-cell acute lymphoblastic leukemia (T-ALL). B-ALL arises from B-lineage lymphoid precursors and constitutes the large majority of childhood ALL, whereas T-ALL originates from immature T-lineage cells and represents a smaller but biologically distinct subgroup [2,3]. Infant ALL, diagnosed before one year of age, represents a rare and clinically distinct subset, accounting for approximately 4% of childhood ALL [4]. Most infant ALL is of B-lineage, and approximately 75% of cases harbor rearrangements involving KMT2A, formerly known as mixed lineage leukemia (MLL) [4]. KMT2A-rearranged disease is characterized by aggressive biology and substantially inferior outcomes compared with childhood ALL overall [4].

The etiology of childhood ALL is multifactorial, reflecting interactions between genetic susceptibility and environmental or developmental exposures [2,5]. Established genetic predisposition includes constitutional chromosome abnormalities such as Down syndrome, as well as inherited leukemia-predisposition syndromes, while germline susceptibility variants in genes involved in lymphoid development and leukemogenesis have also been associated with childhood ALL [2].

Treatment of pediatric ALL consists of prolonged, risk-adapted multiagent therapy designed to eradicate systemic disease while preventing relapse within sanctuary sites [3,6]. Therapy generally includes induction, consolidation/intensification, delayed intensification or re-induction in many protocols, and maintenance, together with central nervous system-directed therapy [6]. Commonly used agents include vincristine, corticosteroids, asparaginase, anthracyclines, methotrexate, mercaptopurine, cyclophosphamide, and cytarabine, with specific combinations and intensities determined by treatment protocol and risk stratification [3]. Treatment intensity is individualized according to clinical characteristics, leukemia genetic and molecular features, and early treatment response, particularly measurable residual disease [3].

While there are many factors that can affect cancer treatment, obesity can influence how chemotherapy is dosed or processed in the body and may impact effectiveness and side effects. Obesity in children and adolescents is commonly defined using age- and sex-specific body mass index (BMI), calculated as weight in kilograms divided by height in meters squared. In the United States, obesity in individuals aged 2-19 years is defined as a BMI at or above the 95th percentile for age and sex, as shown on Centers for Disease Control and Prevention (CDC) growth charts [7]. Obesity can alter body composition, tissue perfusion, plasma protein concentrations, organ size and function, and physiologic processes involved in drug metabolism and elimination [8]. Consequently, pharmacokinetic parameters such as volume of distribution and clearance may differ between children with and without obesity, although these effects are drug-specific [8]. As many pediatric chemotherapy agents are dosed according to body weight or body surface area (BSA), obesity-associated differences in drug disposition may affect systemic exposure, while optimal dosing approaches for many medications in children with obesity remain uncertain [8,9].

Childhood obesity has increased substantially worldwide. A pooled analysis of approximately 129 million individuals demonstrated a marked global increase in childhood and adolescent obesity between 1975 and 2016 [10]. In the United States, obesity prevalence among individuals aged 2-19 years increased from 14.7% in 1999-2000 to 19.2% in 2017-2018, while severe obesity increased from 3.9% to 6.1% during the same period [11]. Thus, the increasing prevalence of childhood obesity makes understanding its potential effects on chemotherapy pharmacokinetics, toxicity, and treatment outcomes increasingly relevant to pediatric oncology.

Given the intensive and prolonged multidrug regimens required to treat pediatric ALL, determining whether obesity influences chemotherapy disposition, toxicity, or therapeutic effectiveness has important clinical implications. This review aims to evaluate the impact of obesity on chemotherapy pharmacokinetics in pediatric ALL, examine associations with treatment-related toxicities, and assess its relationship with clinically important outcomes, including relapse, event-free survival, and overall survival.

## Review

Methods

This narrative literature review examined the influence of obesity on chemotherapy pharmacokinetics, treatment-related toxicity, and clinical outcomes in pediatric ALL. The research team established the scope and principal questions of the review before conducting the literature search. We identified relevant publications through searches of PubMed and Google Scholar. Eligible literature included English-language publications from 2016 onward, with priority given to original human and animal studies. We also used systematic reviews, meta-analyses, and other review articles when they provided relevant background or synthesized the available evidence.

The literature search incorporated combinations of terms related to the disease, exposure, and outcomes of interest, including “pediatric acute lymphoblastic leukemia”, “B-cell acute lymphoblastic leukemia”, “T-cell acute lymphoblastic leukemia”, “obesity”, “body mass index”, “chemotherapy”, “pharmacokinetics”, “drug dosing”, “toxicity”, “treatment-related toxicity”, “relapse”, “event-free survival”, and “overall survival”. We used Boolean operators (AND/OR) to refine literature retrieval. Studies were selected based on their relevance to the predefined objectives, with emphasis on evidence examining obesity in relation to the chemotherapeutic agents used to manage pediatric ALL, chemotherapy-associated adverse effects, and treatment outcomes.

Research team members were assigned sections of the review for independent evaluation, drafting, and subsequent synthesis of the manuscript. Drafted sections underwent internal peer review for scientific accuracy, clarity, and consistency. Two study members then reviewed the completed manuscript, resolving disagreements about interpretation or presentation of evidence through discussion and consensus.

General pharmacokinetics of ALL chemotherapy drugs

Pediatric ALL therapy involves sequential administration of agents with substantially different mechanisms of action, distribution characteristics, metabolic pathways, and routes of elimination [3]. Treatment architecture varies according to leukemia lineage, risk group, molecular characteristics, and treatment protocol [3]. Many contemporary B-ALL protocols include induction, consolidation, interim maintenance (IM), delayed intensification (DI), and maintenance, although specific phases and drug combinations vary by regimen [3]. The current standard of care for B-ALL therapy has also incorporated blinatumomab; in the Children’s Oncology Group (COG) protocol AALL1731, two non-sequential 28-day cycles were incorporated into post-induction therapy for eligible patients with standard-risk B-ALL [12]. T-ALL follows a related but distinct treatment backbone, with COG protocols such as AALL0434 and AALL1231 having evaluated modifications to conventional multiagent ALL therapy; notably, AALL0434 evaluated the addition of nelarabine to frontline therapy, with nelarabine administered during consolidation, delayed intensification, and early maintenance, whereas AALL1231 evaluated the addition of bortezomib during induction and delayed intensification [13,14]. Infant ALL requires separate consideration because treatment protocols may incorporate infant-specific dosing approaches and distinct post-induction treatment blocks rather than necessarily following the conventional IM/DI structure used in older children [4,15].

When protocols use IM1/IM2 or DI1/DI2 designations, drug composition varies by the specific treatment backbone; therefore, information is mapped to agents to these subdivisions only in the context of a defined protocol [3,12,13]. Table 1 summarizes the phases in which major agents are commonly encountered and their principal pharmacologic characteristics.

**Table 1:** Pharmacokinetic characteristics, mechanisms of action, and toxicities of therapeutic agents used in pediatric acute lymphoblastic leukemia. * Drug placement varies according to ALL subtype, risk classification, and treatment protocol. IM1/IM2 and DI1/DI2 terminology and drug composition are not universal. ALL: acute lymphoblastic leukemia; B-ALL: B-cell acute lymphoblastic leukemia; IM: interim maintenance; DI: delayed intensification; ara-CTP: cytosine arabinoside triphosphate; ara-G: arabinosylguanine; BSA: body surface area.

Drug	Common cycle/phase(s)*	Mechanism of action	Pharmacokinetics and metabolism/elimination	Major adverse effects
Vincristine [16,17]	Induction, consolidation, IM, DI, maintenance pulses	Binds beta-tubulin and disrupts microtubule assembly and mitosis	IV administration; extensive tissue distribution; predominantly CYP3A4/3A5-mediated hepatic metabolism, and biliary elimination	Peripheral and autonomic neuropathy, constipation
Dexamethasone/prednisone [18,19]	Induction/DI; protocol-dependent pulses	Glucocorticoid receptor-mediated transcriptional effects promote lymphoblast apoptosis	Prednisone is converted to prednisolone; prednisolone demonstrates substantial interpatient pharmacokinetic variability and concentration-dependent protein binding in children with ALL	Hyperglycemia, infection, hypertension, behavioral effects, osteonecrosis
Pegaspargase/calaspargase pegol [20]	Induction, consolidation, IM, and DI depending on protocol	Enzymatically depletes circulating asparagine required for leukemic protein synthesis	PEGylated *E. coli*-derived asparaginase formulations with prolonged circulating activity; calaspargase contains a more hydrolytically stable linker than pegaspargase, resulting in a longer pharmacokinetic half-life and duration of activity and permitting a longer dosing interval	Hypersensitivity, pancreatitis, thrombosis, hepatotoxicity, hypertriglyceridemia
Daunorubicin/doxorubicin [3,21]	Induction, DI/Intensification	DNA intercalation and topoisomerase II inhibition with generation of reactive oxygen species	Extensive tissue distribution and hepatic metabolism; pediatric doxorubicin clearance demonstrates age-related variability, while individual anthracyclines have distinct pharmacokinetic profiles	Myelosuppression, mucositis, cardiotoxicity
Methotrexate (MTX) [3,22]	Consolidation, IM1/IM2 where applicable, maintenance; intrathecal therapy throughout treatment	Inhibits dihydrofolate reductase and folate-dependent nucleotide synthesis	Intracellular polyglutamation, which prolongs cellular retention; high-dose systemic MTX is predominantly eliminated unchanged through the kidneys, making renal function an important determinant of clearance	Mucositis, myelosuppression, hepatotoxicity, nephrotoxicity, neurotoxicity
6-mercaptopurine (6-MP) [23,24]	Consolidation/IM in selected protocols, maintenance	Purine analogue converted to thioguanine nucleotides that disrupt DNA synthesis	Variable oral bioavailability and complex intracellular activation; TPMT methylates thiopurine metabolites, whereas NUDT15 hydrolyzes active thioguanine triphosphates, limiting their incorporation into DNA	Myelosuppression, hepatotoxicity
Thioguanine (6-TG) [24,25]	Selected DI/intensification phases	Converted to active thioguanine nucleotides that are incorporated into DNA	Intracellular activation and inactivation are influenced by thiopurine metabolic pathways, including TPMT and NUDT15	Myelosuppression, hepatotoxicity, sinusoidal obstruction syndrome
Cyclophosphamide [3,26]	Consolidation, DI/intensification - protocol-specific use in infant regimens	Alkylating prodrug whose active metabolites form DNA cross-links	Requires hepatic CYP-mediated bioactivation, with CYP2B6 among several contributing enzymes; active and inactive metabolites subsequently undergo elimination	Myelosuppression, hemorrhagic cystitis, gonadal toxicity with cumulative exposure
Cytarabine [3,27]	Consolidation, DI/intensification; prominent in some infant regimens	Converted intracellularly to ara-CTP, which inhibits DNA polymerase and DNA synthesis	Rapidly inactivated by cytidine deaminase in the liver, kidney, blood, and other tissues; inactive metabolites are predominantly renally eliminated	Myelosuppression, mucositis, hepatotoxicity; notable for neurotoxicity with higher-dose exposure
Blinatumomab [12,28,29]	Two non-sequential cycles in selected B-ALL protocols	Bispecific CD19/CD3 T-cell engager that redirects T-cells against CD19 positive B-cells	Administered by continuous IV infusion; population pharmacokinetics is described by a one-compartment linear model with first-order elimination; BSA significantly influences clearance, supporting BSA-based dosing in patients weighing less than 45 kg	Cytokine-release syndrome, neurotoxicity, infection
Nelarabine [13,30]	Consolidation, DI, and early maintenance in AALL0434	Prodrug converted to ara-G; intracellular phosphorylation generates ara-GTP, which is incorporated into DNA and inhibits DNA synthesis	Converted to ara-G by adenosine deaminase, followed by intracellular phosphorylation to ara-GTP; ara-G exhibits substantially longer systemic persistence than the parent prodrug	Central and peripheral neurotoxicity, myelosuppression
Bortezomib [14,31]	Induction and DI in AALL1231	Reversibly inhibits the 26S proteasome, disrupting proteasomal protein degradation and promoting apoptosis	IV administration in AALL1231; pediatric population pharmacokinetics demonstrate extensive distribution and multicompartment disposition, with BSA an important determinant of clearance, supporting BSA-based pediatric dosing	Peripheral neuropathy, myelosuppression, gastrointestinal toxicity, infection

The pharmacokinetic diversity of these agents has important implications for systemic exposure. Vincristine depends substantially on CYP3A-mediated metabolism, whereas cyclophosphamide requires CYP-mediated bioactivation to generate cytotoxic metabolites [16,26]. High-dose systemic methotrexate (MTX) provides an important contrast because renal elimination is a major determinant of clearance, and impaired renal function can delay elimination and increase systemic exposure [22]. PEGylated asparaginase preparations exhibit prolonged circulating enzymatic activity, with duration varying by formulation, whereas blinatumomab requires continuous infusion to maintain therapeutic exposure [20,28].

Thiopurines illustrate the importance of intracellular metabolism and pharmacogenomics. Mercaptopurine is enzymatically converted to active thioguanine nucleotides. TPMT-mediated methylation contributes to thiopurine inactivation, whereas NUDT15 hydrolyzes cytotoxic thioguanine triphosphate metabolites, thereby reducing their incorporation into DNA. Reduced-function variants in either gene can substantially increase susceptibility to thiopurine toxicity and require lower tolerated doses [23,24].

Several factors independent of obesity can alter chemotherapy pharmacokinetics. Age and developmental maturation are particularly important in infants because hepatic enzyme activity, renal function, plasma protein binding, and body composition differ from those of older children [15]. Organ dysfunction can also alter drug disposition. Renal impairment is particularly important for high-dose MTX, whereas hepatic dysfunction may influence agents that depend on hepatic metabolism or biliary elimination, including vincristine and cyclophosphamide [16,22,26]. Pharmacogenomic variation provides another source of interpatient variability, most clearly demonstrated by TPMT and NUDT15 during thiopurine therapy [23,24].

Drug-drug interactions are also clinically important. Concomitant azole antifungals can interfere with pathways involved in vincristine disposition and have been associated with increased vincristine neurotoxicity in pediatric patients [17]. Route and duration of administration, concomitant medications, gastrointestinal absorption, developmental physiology, organ function, and interindividual differences in drug-metabolizing enzymes and transporters may contribute to variability in drug disposition [8,15]. Collectively, these sources of pharmacokinetic variability provide the baseline against which the independent effects of obesity on chemotherapeutic agents can be evaluated.

Impact of obesity on changes in pharmacokinetics of ALL chemotherapy drugs

Obesity can alter pharmacokinetics by changing body composition and physiologic processes that affect drug distribution and elimination. Increased adiposity, together with changes in lean body mass and organ size, may influence volume of distribution and clearance, although the direction and magnitude of these effects are drug-specific [8]. Obesity-related renal changes include glomerular hyperfiltration and increased intraglomerular pressure, which may contribute over time to glomerular injury and impaired renal function [32]. Hepatic effects are similarly heterogeneous; in children and adolescents with nonalcoholic steatohepatitis (NASH), Li et al. demonstrated altered cytochrome P450 activity, including significantly reduced CYP2C19 activity, indicating that obesity-associated hepatic disease can modify drug-metabolizing capacity [33].

These physiologic alterations may have different implications for individual ALL therapies. For hepatically metabolized agents such as vincristine and cyclophosphamide, obesity-associated changes in hepatic physiology provide a biologically plausible mechanism for altered drug disposition; however, obesity-specific pharmacokinetic data for these agents in pediatric ALL are limited [8]. Obesity has nevertheless been associated with greater vincristine-induced peripheral neuropathy in children with ALL, although this clinical association does not establish altered vincristine pharmacokinetics as its mechanism [34].

More direct evidence of obesity-associated differences in drug disposition or metabolite exposure exists for MTX, asparaginase, and thiopurines. In 36 children and adolescents receiving high-dose methotrexate (HD-MTX), neither BSA nor body fat percentage was linearly associated with MTX area under the concentration-time curve; however, larger body size and greater body fat were each associated with an approximately two-fold greater risk of delayed elimination at 48 hours despite BSA-adjusted dosing [35]. During re-induction therapy, Panetta et al. found pegaspargase clearance to be 5.3% lower in patients with obesity than in other patients; however, pegaspargase exposure, toxicity, and treatment efficacy did not differ significantly by obesity status [36]. During maintenance therapy, children with extreme obesity had significantly lower erythrocyte thioguanine nucleotide concentrations than children who were underweight or of normal weight, even after adjustment for 6-mercaptopurine (6-MP) dose intensity and adherence; however, these lower metabolite concentrations did not explain the increased relapse risk observed in patients with extreme obesity [37].

Obesity is also associated with several treatment-related toxicities, although these associations should not automatically be attributed to altered pharmacokinetics. In a cohort of 262 children and adolescents with ALL, obesity independently predicted hepatotoxicity and pancreatitis during therapy [38]. Obesity was also independently associated with treatment-related conjugated hyperbilirubinemia during induction and consolidation [39]. Separately, obesity has been associated with greater vincristine-induced peripheral neuropathy [34]. Collectively, these findings support increased attention to treatment-related toxicity in children with obesity, while not establishing increased systemic drug exposure as the mechanism underlying these associations [34,38,39].

Pre-existing obesity and obesity development during treatment may have different implications. In a cohort of 794 children with ALL, overweight or obesity at diagnosis or development of overweight or obesity during induction was not associated with increased treatment-related toxic effects during induction [40]. In contrast, overweight or obesity present at two or more treatment time points was associated with inferior overall and event-free survival and a higher risk of relapse [40]. These findings suggest that persistent overweight or obesity during therapy may carry greater prognostic significance than overweight or obesity identified at an isolated time point, although direct evidence that the timing or duration of obesity produces distinct pharmacokinetic changes remains limited [40].

Obesity-associated fatty liver disease may further complicate ALL pharmacotherapy. The CYP-mediated metabolic alterations described in pediatric NASH may affect ALL agents that depend on hepatic metabolism, although direct studies evaluating how pre-existing NASH modifies the pharmacokinetics of individual ALL drugs are lacking [33]. This may be relevant to ALL agents dependent on hepatic metabolism, although direct studies evaluating how pre-existing NASH modifies the pharmacokinetics of individual ALL drugs are lacking. Asparaginase therapy itself is associated with clinically important hepatotoxicity, creating an additional concern in patients with underlying hepatic dysfunction [20]. Thus, NASH may be an additional consideration beyond BMI alone when evaluating hepatic function during ALL therapy, although its effects on the pharmacokinetics of specific ALL agents remain unclear.

Management should emphasize drug-specific monitoring rather than empirical dose modification based solely on obesity. Serial MTX concentrations following HD-MTX are required to identify delayed elimination, while serum asparaginase activity can be used to assess therapeutic asparaginase exposure [20,35]. Importantly, the modest reduction in pegaspargase clearance observed with obesity did not translate into significant differences in exposure, toxicity, or efficacy, and Panetta et al. did not support empirical pegaspargase dose capping on the basis of obesity in patients treated with a regimen similar to St. Jude Total Therapy Study XVI (Total XVI) [36]. As obesity-related pharmacokinetic effects vary among agents and remain incompletely characterized, available evidence does not support assuming that obesity uniformly increases or decreases chemotherapy clearance [8].

Chemotherapy dosing considerations in pediatric ALL patients with obesity

Pediatric ALL therapy uses protocol-defined, phase-specific multidrug regimens, with many systemic agents dosed according to BSA [6,41]. In children with obesity, determining whether total body weight, ideal body weight, adjusted body weight, or another body-size descriptor should inform drug dosing remains challenging because obesity alters body composition and can affect drug distribution and clearance differently among medications [8,9]. A systematic review by the International Society of Pediatric Oncology (SIOP) Nutrition Network identified substantial heterogeneity in definitions of nutritional status, body-composition measures, chemotherapy dosing strategies, and pharmacokinetic analyses across pediatric oncology studies; consequently, the available evidence was insufficient to formulate chemotherapy dosing recommendations based on nutritional status or body composition [41].

Current evidence does not establish a consistent pharmacokinetic rationale for empirical chemotherapy dose reduction or altered treatment frequency based solely on obesity [8,41]. Available pediatric oncology studies have used heterogeneous body-size metrics and dosing approaches, and current evidence is insufficient to identify a preferred alternative body-size metric for chemotherapy dosing in children with obesity [41]. Therefore, obesity-associated pharmacokinetic differences should be evaluated on a drug-specific basis.

For HD-MTX, available evidence does not establish a need for pre-emptive dose reduction solely because of obesity; serial MTX concentrations should instead be used to identify delayed elimination and guide subsequent management [35]. Similarly, the modest obesity-associated reduction in pegaspargase clearance reported by Panetta et al. did not establish a pharmacokinetic basis for empirical dose reduction solely because of obesity [36]. Serum asparaginase activity can directly assess therapeutic asparaginase exposure when monitoring is clinically or protocol-indicated [20]. Table 2 summarizes dosing and monitoring considerations for selected agents used in pediatric ALL.

**Table 2:** Dosing and monitoring considerations for selected ALL therapies in pediatric patients with obesity. ALL: acute lymphoblastic leukemia; MTX: methotrexate.

Agent	Dosing and monitoring considerations
Vincristine	Obesity has been associated with greater vincristine-induced peripheral neuropathy, supporting careful toxicity surveillance in affected patients [34].
High-dose methotrexate (HD-MTX)	Greater body size and body fat may increase the risk of delayed elimination, supporting serial MTX concentration monitoring rather than empirical obesity-based reduction of the initial dose [35].
Pegaspargase	Modestly reduced clearance in patients with obesity has not been shown to produce clinically significant differences in exposure, toxicity, or efficacy; obesity alone therefore does not establish a pharmacokinetic rationale for empirical dose reduction [36]. Serum asparaginase activity monitoring may be used when clinically or protocol indicated [20].
6-mercaptopurine (6-MP)	Obesity has been associated with lower thioguanine nucleotide concentrations during maintenance, but these differences did not explain the increased risk of relapse seen in patients with extreme obesity [37]. TPMT and NUDT15 status provide an established basis for genotype-guided thiopurine dose modification [24].
Other cytotoxic agents	Current pediatric evidence is insufficient to support universal obesity-specific alterations in chemotherapy dose or frequency; dosing should follow drug- and protocol-specific criteria [41].

During maintenance, the lower erythrocyte thioguanine nucleotide concentrations observed among patients with extreme obesity do not support an empirical, obesity-based 6-MP dose reduction [37]. In contrast, TPMT and NUDT15 metabolizer status provides an established pharmacogenetic basis for thiopurine starting-dose modification [24].

Importantly, obesity itself should be distinguished from obesity-associated organ dysfunction or treatment-related toxicity. Therefore, it is important to consider hepatic or renal dysfunction, treatment-related toxicity, or delayed drug elimination separately from BMI when applying agent- and protocol-specific criteria for treatment modification. Although obesity has been associated with several treatment-related toxicities in pediatric ALL, these associations do not independently demonstrate that reducing chemotherapy doses solely because of obesity improves safety [34,38,39].

Until obesity-specific dosing strategies are prospectively validated, chemotherapy dosing should remain drug- and protocol-specific and incorporate the prescribed body-size metric, organ function, pharmacogenetics, validated therapeutic drug monitoring where available, and observed treatment-related toxicity [8,24,41]. Prospective studies incorporating standardized nutritional assessment and body-composition measures are needed to determine whether specific agents or patient subgroups benefit from obesity-specific dosing approaches [41].

Discussion

Obesity represents an important therapeutic consideration in pediatric ALL, where treatment requires prolonged exposure to multiple agents with markedly different pharmacokinetic properties. Available evidence demonstrates that obesity can modify physiologic determinants of drug disposition, including body composition, hepatic metabolism, and renal function; however, the effects of obesity on drug disposition are drug-specific, and evidence characterizing these effects for individual chemotherapeutic agents remains limited [8,41]. Consequently, theoretical alterations in drug distribution or clearance should not be assumed to produce clinically meaningful differences in systemic exposure or independently justify empirical dose modification [8,41].

Available drug-specific evidence reflects this difference. Greater body size and body fat have been associated with delayed HD-MTX elimination without a corresponding linear increase in systemic exposure [35]. During re-induction, pegaspargase clearance was modestly lower in patients with obesity, but this did not produce significantly greater asparaginase exposure or clear differences in treatment outcomes [36]. Similarly, extreme obesity has been associated with lower erythrocyte thioguanine nucleotide concentrations during 6-MP maintenance, yet these differences did not account for the increased relapse risk observed in these patients [37]. Collectively, these findings indicate that a simple model in which obesity uniformly increases or decreases chemotherapy exposure cannot explain the relationship between obesity and ALL outcomes [35-37].

Obesity nevertheless appears clinically relevant to treatment-related toxicity. Associations have been demonstrated between obesity and hepatotoxicity, pancreatitis, conjugated hyperbilirubinemia, and vincristine-induced peripheral neuropathy [34,38,39]. Importantly, these findings demonstrate associations with toxicity rather than establishing altered pharmacokinetics as the underlying mechanism. Obesity-associated hepatic disease may provide an additional source of variability because pediatric NASH has been associated with alterations in CYP-mediated metabolism [33]. Therefore, BMI, obesity-associated organ dysfunction, treatment-related toxicity, and measured drug exposure should be considered related by distinct clinical variables.

The relationship between obesity and survival outcomes is particularly important. In a cohort study of 794 children with ALL, overweight or obesity at diagnosis or new-onset overweight or obesity during induction was not associated with increased treatment-related toxic effects during induction; however, overweight or obesity present at two or more treatment time points was associated with inferior overall and event-free survival and a higher risk of relapse [40]. Wadhwa et al. similarly demonstrated that extreme obesity during maintenance was associated with an increased risk of relapse, whereas relapse risk among patients classified as overweight or obese, but not extremely obese, was comparable to that of underweight or normal-weight patients [37]. Together, these findings suggest that the relationship between BMI-defined weight status and ALL outcomes may differ by the degree and persistence of overweight or obesity rather than by its presence at a single time point. The mechanisms underlying these associations remain unclear and cannot currently be attributed exclusively to altered chemotherapy pharmacokinetics.

These findings have several implications for clinical practice. Current evidence is insufficient to support routine modification of chemotherapy dosing solely on the basis of obesity [8,41]. Therefore, without validated obesity-specific dosing strategies, obesity alone should not guide empirical chemotherapy dose modification. Serial MTX concentrations following HD-MTX and serum asparaginase activity monitoring show how measured drug disposition or activity can inform management more directly than BMI alone [20,35]. Longitudinal assessment of BMI throughout therapy may also be clinically informative because the duration and degree of overweight or obesity appear more strongly associated with adverse outcomes than overweight or obesity identified at an isolated time point [37,40].

However, evidence gaps remain. Pediatric studies examining obesity-specific chemotherapy pharmacokinetics are limited and heterogeneous in their definitions of nutritional status, assessment of body composition, dosing strategies, and pharmacokinetic analyses, preventing definitive recommendations regarding alternative dosing approaches for children with obesity [41]. Future prospective studies should incorporate longitudinal measures of BMI and body composition alongside agent-specific pharmacokinetic and pharmacodynamic measurements, treatment adherence, pharmacogenetics, organ function, toxicity, and survival outcomes. Such studies may identify patient subgroups who could benefit from individualized dosing strategies while avoiding empirical dose reductions that could compromise chemotherapeutic exposure. Until additional evidence is available, obesity should be recognized as a clinical factor associated with treatment-related toxicity and outcomes, but not as an independent indication for generalized chemotherapy dose reduction.

## Conclusions

Obesity represents an increasingly relevant consideration in pediatric ALL, but its effects on chemotherapy pharmacokinetics are complex and drug-specific. Available evidence suggests obesity may influence the disposition of selected agents and is associated with treatment-related toxicities and adverse outcomes, including increased relapse risk and inferior survival in certain populations. However, these associations cannot currently be attributed solely to altered chemotherapy exposure. Existing evidence does not support generalized chemotherapy dose reduction based on obesity alone. Instead, treatment should remain protocol- and drug-specific, incorporating organ function, pharmacogenetics, therapeutic drug monitoring when available, and observed toxicity. Longitudinal assessment of weight status may provide additional clinical information beyond measurements obtained at diagnosis alone. Future prospective studies integrating standardized measures of BMI and body composition with pharmacokinetic, pharmacodynamic, toxicity, and survival outcomes are needed to clarify the mechanisms underlying obesity associated risk and determine whether specific patient subgroups may benefit from individualized dosing strategies.
